# Impact of red blood cell transfusion and transfusion strategies on clinical outcomes in extremely low gestational age neonates

**DOI:** 10.3389/fped.2026.1729671

**Published:** 2026-05-28

**Authors:** Xuejing Liu, Junling Ma, Fangrui Ding

**Affiliations:** 1 Department of Neonatology, Tianjin Central Hospital of Gynecology Obstetrics, Tianjin, China; 2 Tianjin Key Laboratory of Human Development and Reproductive Regulation, Tianjin Central Hospital of Gynecology Obstetrics, Tianjin, China

**Keywords:** BPD (bronchopulmonary dysplasia), complications, extremely low gestational age neonates, red blood cell transfusion, restrictive transfusion

## Abstract

**Objective:**

To investigate the effects of red blood cell transfusions and transfusion strategies on clinical outcomes in extremely low gestational age neonates (ELGANs).

**Methods:**

This retrospective cohort study enrolled 545 ELGANs with gestational age <28 weeks admitted between 2012 and 2024. Infants were divided into transfusion and non-transfusion groups. Tra nsfused infants were further classified into restrictive, liberal, and relatively liberal transfusion groups according to transfusion thresholds, and intergroup differences in clinical outcomes were analyzed. Multivariate logistic regression was performed to analyze the independent association between transfusion and outcomes, adjusted for treatment era (2012–2019 vs. 2020–2024), perinatal factors, postnatal illness severity, and treatment-related variables. Primary outcomes were bronchopulmonary dysplasia (BPD) and survival without severe complications. Secondary outcomes included severe retinopathy of prematurity (ROP), necrotizing enterocolitis (NEC), severe brain injury, and death.

**Results:**

A total of 545 ELGANs were included, with a median gestational age of 26.5 (IQR 25.6–27.2) weeks. Among them, 346 infants (63.5%) received at least one transfusion, with a total of 798 transfusion episodes and a median number of transfusions of 2 (IQR 1–3). Of the transfused infants, 82 (25.0%) were in the restrictive transfusion group, 138 (39.9%) in the liberal transfusion group, and 92 (26.6%) in the relatively liberal transfusion group. An increased number of transfusions was an independent risk factor for BPD (adjusted OR = 1.88, 95% CI: 1.48–2.40, *p* < 0.001) and was independently associated with a lower rate of survival without severe complications (adjusted OR = 0.75, 95% CI: 0.63–0.91, *p* = 0.003). Meanwhile, a higher transfusion number was independently associated with lower risks of severe brain injury (adjusted OR = 0.62, 95% CI: 0.46–0.84, *p* = 0.002) and mortality (adjusted OR = 0.66, 95% CI: 0.53–0.83, *p* < 0.001). Transfusion number was not independently associated with severe ROP or NEC. The restrictive transfusion group had significantly fewer transfusions than the relatively liberal transfusion group, with no significant differences in BPD, survival without severe complications, or other adverse outcomes compared with the other two groups.

**Conclusions:**

In ELGANs, an increased number of transfusions is independently associated with a higher risk of BPD and lower survival without severe complications. A restrictive transfusion strategy safely reduces transfusion exposure without increasing the risk of major adverse outcomes and is recommended in clinical practice.

## 1 Introduction

Anemia of prematurity is a common clinical problem in neonatal intensive care units (NICU). Its pathogenesis involves multifactorial interactions, including inadequate fetal iron stores, low erythropoietin level, a shortened life span for preterm red blood cells, rapid increase in somatic growth, low circulating blood volume, proportionately high phlebotomy rates, and critical illnesses (1). Red blood cell transfusion (RBCT) is an effective therapeutic intervention. Hereafter, it will be referred to simply as transfusion. Significant variations in transfusion rates exist across centers due to differences in transfusion guidelines and adherence, with rates inversely correlating with both gestational age (GA) and birth weight (BW) (2–4). Reported transfusion rates among extremely low birth weight infants range from 64% to over 90% (2, 4). Multicenter studies in China indicated that extremely low gestational age neonates (ELGANs, defined as infants born at <28 weeks' gestation) have transfusion rates as high as approximately 80% (3), highlighting the particular dependence of this high-risk population on transfusion interventions. However, transfusions may increase the risk of adverse reactions and events. Data from the UK's Serious Hazards of Transfusion (SHOT) scheme (5) revealed a significant age gradient in the incidence of adverse reactions and events: 37 per 100,000 in infants less than 12 months, 18 per 100,000 in children less than 18 years, and 13 per 100,000 in adults, suggesting neonates may possess heightened susceptibility. Clinical studies indicated that transfusions may contribute to adverse outcomes including bronchopulmonary dysplasia (BPD) (3, 6), retinopathy of prematurity (ROP) (3, 7), necrotizing enterocolitis (NEC) (8), severe intraventricular hemorrhage (IVH) (9), and neurodevelopmental impairment (NDI) (10). This double-edged effect, balancing therapeutic benefit against potential risk, necessitates a thorough risk-benefit assessment for clinical decisions.

In recent years, the restrictive transfusion strategy has garnered significant attention due to its potential to reduce donor exposure. The two pivotal randomized clinical trials—the Transfusion of Prematures (TOP) trial (4) and the Effects of Transfusion Thresholds on Neurocognitive Outcomes of Extremely Low-Birth-Weight Infants (ETTNO) trial (11)—demonstrated no significant differences in short- or long-term outcomes between the restrictive and liberal transfusion groups. Current research in China primarily focuses on the broader preterm infant population, with limited data available for the high-risk subgroup of ELGANs. This study aims to investigate the impact of transfusion and different transfusion strategies on clinical outcomes in ELGANs through retrospective analysis, thereby providing an evidence-based foundation for clinical decision-making.

## 2 Materials and methods

### 2.1 Study design and participants

We conducted a retrospective study of preterm infants with a GA < 28 weeks admitted to the NICU at Tianjin Central Hospital of Gynecology Obstetrics within 24 h of birth between January 2012 and December 2024. Exclusion criteria: (1) length of hospital stay <3 days; (2) major anomalies (e.g., chromosomal anomalies, cyanotic heart defects, etc.); (3) cases with incomplete data due to treatment withdrawal or hospital transfer.

Infants were divided into transfusion and non-transfusion groups, and transfused infants were further reclassified at the episode level into three groups: the restrictive transfusion group [all pre-transfusion hemoglobin (Hb) levels below the threshold], the liberal transfusion group (all pre-transfusion Hb levels at or above the threshold), and the relatively liberal transfusion group (a mixed pattern with some pre-transfusion Hb levels above and some below the threshold). The thresholds, varied on the basis of postnatal week and respiratory support needs, were established according to the 2024 guidelines of the Neonatal Transfusion Network (12) (detailed in Table 1).

**Table 1:** Restrictive transfusion thresholds recommended by the guidelines.

Transfusion threshold	Respiratory support^a^	No or minimal respiratory support
Hb-based threshold, g/L		
Postnatal wk 1	110	100
Postnatal wk 2	100	85
≥Postnatal wk 3	90	70

a Respiratory support was defined as invasive mechanical ventilation, continuous positive airway pressure or noninvasive intermittent positive pressure ventilation, or nasal cannula flow rate of 1 L/min or greater.

### 2.2 Data collection

General clinical data included: (1) Demographic factors: GA, BW, sex, multiple pregnancy (vs. singleton pregnancy), mode of delivery (cesarean vs. vaginal), and 5-min Apgar score. (2) Maternal factors: maternal age, antenatal corticosteroid use, premature rupture of membranes (PROM), gestational diabetes mellitus (GDM), and hypertensive disorders of pregnancy (HDP). (3) Variables related to transfusion: number of transfusions, postnatal age at first transfusion, pre-transfusion Hb. (4) Neonatal illness severity and clinical variables: pulmonary hemorrhage, sepsis, hemodynamically significant patent ductus arteriosus (hsPDA), severe anemia, Score for Neonatal Acute Physiology Perinatal Extension-II (SNAPPE-II) score, breastfeeding, and respiratory support use.

All ELGANs receiving transfusions were administered a volume of 15–20 mL/kg. Due to the extended duration of the study, transfusion practices were guided by both domestic textbook recommendations and international transfusion guidelines. However, as these guidelines underwent updates over time, no single unified transfusion standard was applied throughout the entire research period.

### 2.3 Outcomes and definitions

Primary outcomes included BPD and survival without severe complications. Secondary outcomes included severe brain injury, severe ROP, NEC (Bell's stage ≥Ⅱ), and death.

BPD was defined as requiring respiratory support at 36 weeks postmenstrual age or discharge, whichever occurred first (13). ROP was classified according to the International Classification (14), with severe ROP defined as stage ≥3 or requiring treatment. Severe brain injury was characterized by either IVH ≥ grade 3 based on Papile's criteria (15) or periventricular leukomalacia (PVL). NEC was classified according to Bell's criteria (16). Survival without severe complications was defined as survival to discharge without BPD, NEC, severe ROP or severe brain injury. HsPDA was defined using a combination of clinical and echocardiographic criteria (17). Illness severity was stratified using the SNAPPE-II score: infants with a score ≥40 were categorized as critically ill, and those with a score <40 as non-critically ill, as previously validated (18). Severe anemia was defined as a hemoglobin level <80 g/L according to previous studies (19).

### 2.4 Statistical analysis

Statistical analyses were performed using SPSS 29.0. Categorical variables were presented as *n* (%) and compared using the Chi-square test or Fisher's exact test. Non-normally distributed continuous variables were expressed as median (interquartile range, IQR) and compared using the Mann–Whitney *U-*test (two groups) or Kruskal-Wallis *H-*test (three or more groups). For comparisons among three transfusion strategy groups, the Kruskal-Wallis test with Bonferroni-corrected pairwise *post hoc* comparisons was used for non-normally distributed continuous variables. Categorical variables were compared using *z*-tests for column proportions. Bonferroni correction was applied to pairwise comparisons to control the family-wise error rate, with an adjusted significance level set at *α* = 0.05/3 ≈ 0.0167. Trends across groups were assessed using the Cochran–Armitage test for binary variables and the Jonckheere–Terpstra test for continuous variables. For missing data, complete-case analysis was used without imputation. Infants who died before 36 weeks postmenstrual age were excluded from the BPD analysis, and infants without standardized ROP screening were excluded from the ROP analysis. Multivariate logistic regression models were constructed to evaluate independent associations between transfusion-related indicators and clinical outcomes. Variables considered clinically important and those for which a statistically significant difference (*p* < 0.10) was observed in the univariate analysis were included in the multivariate models. Given multiple outcomes being tested, the Bonferroni correction was applied to control for type I error, with the significance level set at *α* = 0.05/6 = 0.0083. A two-sided *p* < 0.0083 was considered statistically significant.

## 3 Results

### 3.1 Baseline characteristics and transfusion profile

**Figure 1:**
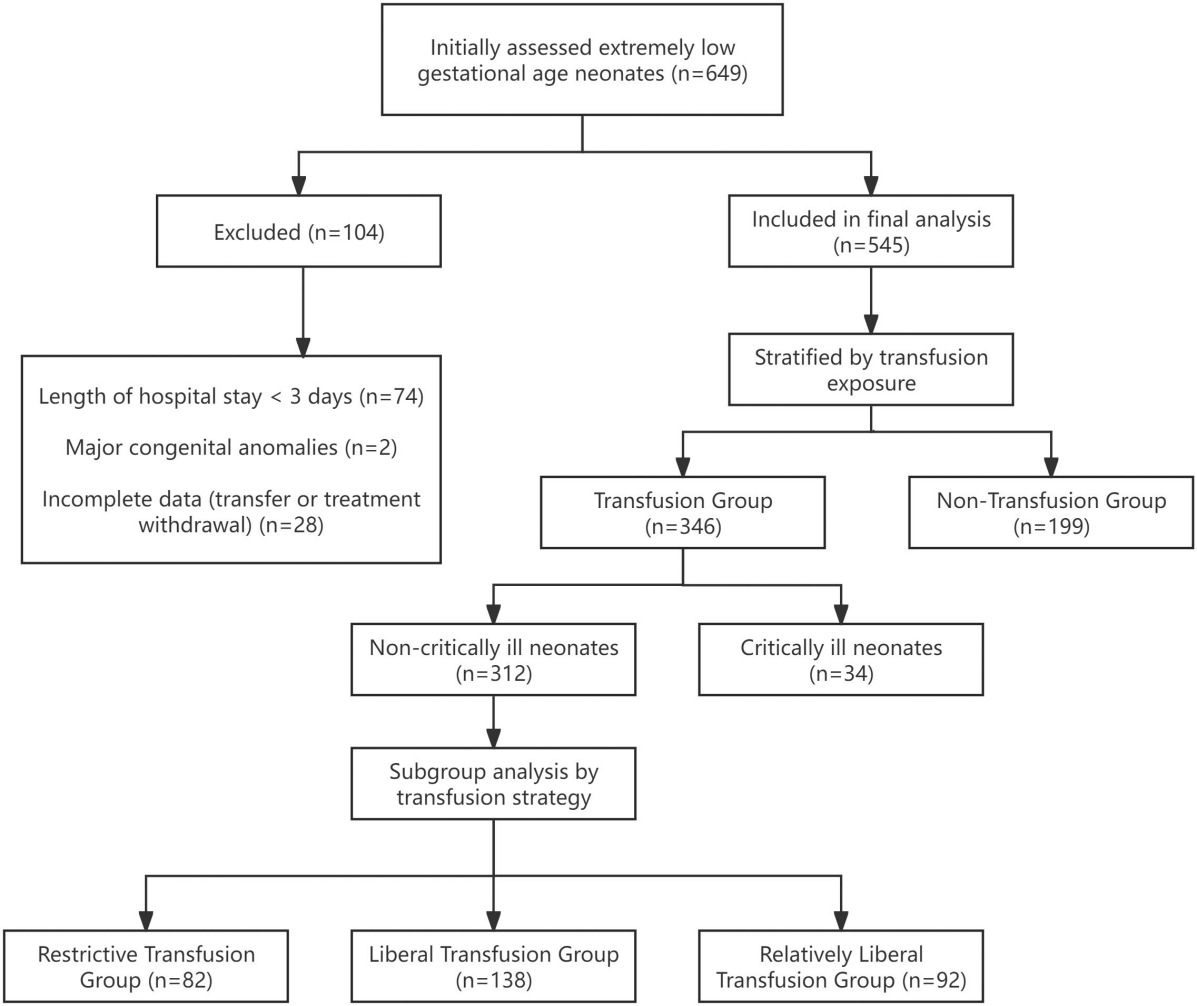
Flowchart of participants inclusion. A total of 649 Extremely Low Gestational Age Neonates were initially assessed, with 104 excluded based on predefined criteria. The remaining 545 infants were included in the final analysis and stratified by transfusion exposure into the Transfusion Group (*n* = 346) and the Non-Transfusion Group (*n* = 199). The Transfusion Group was further divided into Critically Ill Neonates (*n* = 34) and Non-Critically Ill Neonates (*n* = 312). Subgroup analysis by transfusion strategy was performed among the non-critically ill neonates, yielding the Restrictive Transfusion Group (*n* = 82), Liberal Transfusion Group (*n* = 138), and Relatively Liberal Transfusion Group (*n* = 92).

A total of 649 ELGANs were admitted during the study period. After applying the exclusion criteria, 104 infants were excluded, leaving 545 infants for final analysis (Figure 1). The cohort had a median GA of 26.5 (IQR 25.6–27.2) weeks and median BW of 920 (IQR 810–1,020) g, with 301 males (55.2%) and 244 females (44.8%). Overall, 346 infants (63.5%) received at least one transfusion. The median number of transfusions was 2 (IQR 1–3), with a median postnatal age at first transfusion of 30 (IQR 21–41) days. Of the transfused infants, 93 (26.9%) met criteria for the restrictive transfusion group. Lower GA and BW were significantly associated with earlier postnatal age at first transfusion, higher transfusion rates, and greater number of transfusions (*p* < 0.001) (Table 2).

**Table 2:** Transfusion situations in different groups of GA and BW.

GA/BW category	*n* (%)	transfusion, *n* (%)	postnatal age at first transfusion, median (IQR), days	number of transfusions, median (IQR), *n*	restrictive transfusion, *n* (%)
GA					
≤24 w	60 (11.0)	48 (80.0)	15 (10–27)	3 (1–6)	11 (22.9)
25 w	98 (18.0)	79 (80.6)	27 (20–34)	2 (1–3)	24 (30.4)
26 w	162 (29.7)	110 (67.9)	33 (25–45)	2 (1–3)	33 (30.0)
27 w	225 (41.3)	109 (48.4)	34 (25–49)	1 (1–2)	25 (22.9)
*χ*^2^/*Z*	–	37.96	6.90	−4.85	0.15
*P*	–	<0.001	<0.001	<0.001	0.702
BW					
<750 g	74 (13.6)	64 (86.5)	25 (12–30)	3 (1–5)	14 (21.9)
750–1,000 g	308 (56.5)	196 (63.6)	29 (21–37)	2 (1–3)	56 (28.6)
≥1,000 g	163 (29.9)	86 (52.8)	39 (27–50)	1 (1–2)	23 (26.7)
*χ*^2^/*Z*	–	23.04	5.85	−4.91	0.33
*p*	–	<0.001	<0.001	<0.001	0.568
Total	545	346 (63.5)	30 (21–41)	2 (1–3)	93 (26.9)

The Cochran-Armitage or Jonckheere–Terpstra test was used for trend testing; –: No data; GA, gestational age; BW, birth weight.

Among 545 infants, a total of 798 transfusion episodes were recorded, of which 363 episodes (45.5%) were below-threshold transfusions. All transfusions administered during Weeks 1 and 2 occurred in infants requiring varying levels of respiratory support, with no significant differences in pre-transfusion Hb levels across respiratory support strata (*p* > 0.05). In contrast, significant differences in pre-transfusion Hb levels were observed across respiratory support levels for transfusions administered at ≥3 weeks of age (*p* < 0.001) (Table 3).

**Table 3:** Comparison of blood transfusion situations under different postnatal age and respiratory support states.

Postnatal age	Variable	IMV	NIV^a^	No or minimal respiratory support^b^	*χ²*/*Z*	*p*
Postnatal wk 1	*n*	31	6	0		
	restrictive transfusion, *n* (%)	17 (54.8)	3 (50)	–	–	1.000
	pre-transfusion Hb, median (IQR), g/L	106 (81–113)	106 (85–120)	–	−0.43	0.665
Postnatal wk 2	*n*	20	6	0		
	restrictive transfusion, *n* (%)	9 (45.0)	5 (83.3)	–	–	0.170
	pre-transfusion Hb, median (IQR), g/L	103 (90–108)	94 (80–101)	–	−0.98	0.330
≥Postnatal wk 3	*n*	88	472	175		
	restrictive transfusion, *n* (%)	37 (42.0)	291 (61.7)	1 (0.6)	192.94	<0.001
	pre-transfusion Hb, median (IQR), g/L	92 (84–98)	87 (81–92)	84 (80–89)	14.95	<0.001

a Includes continuous positive airway pressure, noninvasive intermittent positive pressure ventilation, and nasal cannula flow rate ≥1 L/min.

b No respiratory support or nasal cannula flow rate <1 L/min. –, No data; IMV, invasive mechanical ventilation; NIV, non-invasive ventilation; Hb, hemoglobin.

### 3.2 Relationship between transfusion and clinical characteristics

Compared with the non-transfusion group, the transfusion group had significantly lower GA, BW, and 5-minute Apgar score (all *p* < 0.05). The proportion of severe anemia, pulmonary hemorrhage, sepsis, hsPDA, and critically ill status were significantly higher in the transfusion group (all *p* < 0.05). There were no significant differences in sex, multiple pregnancy, cesarean delivery, PROM, GDM, HDP, or breastfeeding rate between the two groups (all *p* > 0.05). In addition, the transfusion group had significantly longer length of hospital stay, duration of invasive mechanical ventilation (IMV), and total respiratory support (all *p* < 0.001). The transfusion group had a significantly higher incidence of BPD and a significantly lower rate of survival without severe complications (all *p* < 0.001). For secondary outcomes, the transfusion group had higher rates of severe ROP and NEC (both *p* < 0.05), with no significant differences in severe brain injury or mortality (both *p* > 0.05). See Table 4 for details.

**Table 4:** Comparison of clinical characteristics between the transfusion group and the non-transfusion group.

Variable	non-transfusion	transfusion	*χ²*/*Z*	*p*
	(*n* = 199)^a^	(*n* = 346)^a^		
GA, median (IQR), week	27.1 (26.3–27.4)	26.3 (25.4–27.1)	−6.48	<0.001
BW, median (IQR), g	960 (860–1,080)	880 (780–993)	−5.25	<0.001
5-min Apgar score, median (IQR), *n*	8 (7–9)	8 (7–8)	−2.91	0.004
Male, *n* (%)	106 (53.3)	195 (56.4)	0.49	0.485
Maternal age, median (IQR), year	31 (28–35)	32 (29–36)	−1.95	0.051
Multiple pregnancy, *n* (%)	63 (31.7)	122 (35.3)	0.73	0.393
Cesarean delivery, *n* (%)	35 (17.6)	57 (16.5)	0.11	0.738
Antenatal corticosteroids, *n* (%)	119 (59.8)	253 (73.1)	10.35	0.001
PROM, *n* (%)	48 (24.1)	89 (25.7)	0.17	0.678
GDM, *n* (%)	29 (14.6)	68 (19.7)	2.23	0.135
HDP, *n* (%)	13 (6.5)	23 (6.6)	0.003	0.959
Breastfeeding, *n* (%)	152 (76.4)	288 (83.2%)	3.82	0.051
IMV, *n* (%)	35 (17.6)	136 (39.3)	27.68	<0.001
Severe anemia, *n* (%)	4 (2.0%)	128 (37.0)	84.25	<0.001
Pulmonary hemorrhage, *n* (%)	7 (3.5)	57 (16.5)	20.46	<0.001
Sepsis, *n* (%)	26 (13.1)	125 (36.1)	33.55	<0.001
hsPDA, *n* (%)	30 (15.1)	91 (26.3)	9.22	0.002
Critically ill, *n* (%)	10 (5.0)	34 (9.8)	3.92	0.048
BPD, *n* (%)	10 (6.1) [*n* = 163]	109 (37.3) [*n* = 292]	52.70	<0.001
Survival without severe complications, *n* (%)	128 (64.3)	129 (37.3)	37.07	<0.001
Severe brain injury, *n* (%)	20 (10.1)	30 (8.7)	0.29	0.591
Severe ROP, *n* (%)	22 (13.3) [*n* = 165]	71 (23.5) [*n* = 302]	6.93	0.008
NEC, *n* (%)	4 (2.0)	37 (10.7)	13.69	<0.001
Death, *n* (%)	37 (18.6)	65 (18.8)	0.003	0.956
Length of hospital stay, median (IQR), days	70 (56–78)	85 (70–101)	−8.33	<0.001
Duration of IMV, median (IQR), days	0 (0–0)	0 (0–6)	−5.87	<0.001
Duration of total respiratory support, median (IQR), days	30 (15–42)	52 (36–71)	−10.24	<0.001

a The number of cases varies due to outcome-specific exclusion during data analysis. Denominators are as specified in column heads unless otherwise indicated. GA, gestational age; BW, birth weight; PROM, premature rupture of membranes; GDM, gestational diabetes mellitus; HDP, hypertensive disorders of pregnancy; hsPDA, hemodynamically significant patent ductus arteriosus; BPD, bronchopulmonary dysplasia; ROP, retinopathy of prematurity; NEC, necrotizing enterocolitis; IMV, invasive mechanical ventilation.

### 3.3 Comparison of clinical characteristics and outcomes between two treatment eras

To evaluate potential temporal changes during the study period, we compared clinical practices and outcomes between infants admitted in 2012–2019 (*n* = 286) and 2020–2024 (*n* = 259). Compared with the earlier era, infants in the 2020–2024 era had a significantly higher rate of transfusion, a greater number of transfusions, higher use of IMV, an increased rate of combined continuous positive airway pressure (CPAP) and high-flow nasal cannula (HFNC) use, and longer duration of total respiratory support (all *p* < 0.05). The duration of IMV showed a marginally significant difference between groups (*p* = 0.051). The rate of BPD was significantly higher in the 2020–2024 group, while the rate of survival without severe complications was significantly lower (all *p* < 0.05). No significant differences were observed in severe brain injury, severe ROP, NEC, or mortality between the two eras (all *p* > 0.05). These findings suggest notable temporal changes in clinical practice and disease burden over the 13-year study period. See Table 5 for details.

**Table 5:** Comparison of clinical practices and outcomes between two treatment eras (2012–2019 vs. 2020–2024).

Variable	2012–2019	2020–2024	χ²/Z	*p*
	(*n* = 286)	(*n* = 259)		
transfusion, *n* (%)	143 (50.0)	203 (78.4)	47.22	<0.001
number of transfusions, median (IQR), *n*	1 (1–2)	2 (1–4)	−6.18	<0.001
IMV, *n* (%)	79 (27.6)	92 (35.5)	3.94	0.047
NIV			32.62	<0.001
CPAP, *n* (%)	262 (91.6)	212 (81.9)		
CPAP + HFNC, *n* (%)	9 (3.1)	43 (16.6)		
Duration of IMV, median (IQR), days	0 (0, 1)	0 (0, 4)	−1.95	0.051
Duration of total respiratory support, median (IQR), days	37 (21–51)	49 (35–68)	−6.35	<0.001
BPD, *n* (%)	47/236 (19.9)	72/219 (32.9)	9.88	0.002
Survival without severe complications, *n* (%)	155 (54.2)	102 (39.4)	11.97	<0.001
Severe brain injury, *n* (%)	18 (6.3)	23 (8.9)	1.31	0.253
Severe ROP, *n* (%)	40/241 (16.6)	53/226 (23.5)	3.44	0.064
NEC, *n* (%)	25 (8.7)	25 (9.7)	0.14	0.713
Death, *n* (%)	55 (19.2)	47 (18.1)	0.11	0.746

IMV, invasive mechanical ventilation; NIV, non-invasive ventilation; CPAP, continuous positive airway pressure; HFNC, high-flow nasal cannula; BPD, bronchopulmonary dysplasia; ROP, retinopathy of prematurity; NEC, necrotizing enterocolitis.

### 3.4 Association between the number of transfusions and the outcomes

Covariates were selected based on clinical plausibility, prior literature, and results from univariate analyses (*p* < 0.10). Variables known or suspected to be associated with both the exposure (number of transfusions) and the outcomes (BPD, survival without severe complications, severe brain injury, severe ROP, NEC, and death) were included in multivariate models to control for potential confounding. The covariates included GA, birth weight, 5-minute Apgar score, antenatal corticosteroid use, maternal age, multiple pregnancy, cesarean delivery, GDM, HDP, pulmonary hemorrhage, sepsis, hsPDA, severe anemia, breastfeeding status, and IMV use. Furthermore, considering the long study period from 2012 to 2024 during which transfusion thresholds and clinical management strategies evolved over time, treatment era was incorporated as a covariate in all regression models to account for temporal heterogeneity and minimize time-related confounding. Notably, given its clinical relevance and inclusion in the composite definition of survival without severe complications, NEC was included as a covariate in all models for the other relevant outcomes. Bonferroni correction was applied to address multiple testing. To minimize reverse causation and time-dependent bias, only transfusions and covariates that occurred before the onset of each outcome were included in the corresponding models; events occurring after outcome diagnosis were excluded.

After full adjustment, the number of transfusions was independently associated with an increased risk of BPD (adjusted OR = 1.88, 95% CI: 1.48–2.40, *p* < 0.001) and a lower likelihood of survival without severe complications (adjusted OR = 0.75, 95% CI: 0.63–0.91, *p* = 0.003). Both associations remained statistically significant after Bonferroni correction (*p* < 0.0083). For secondary outcomes, a higher number of transfusions was independently associated with a lower risk of severe brain injury (adjusted OR = 0.62, 95% CI: 0.46–0.84, *p* = 0.002) and a lower risk of mortality (adjusted OR = 0.66, 95% CI: 0.53–0.83, *p* < 0.001). Both associations also remained statistically significant after Bonferroni correction (*p* < 0.0083). No significant independent associations were found between transfusion number and severe ROP or NEC (all *p* > 0.05). Details are presented in Table 6.

**Table 6:** Association between the number of transfusions and the primary outcomes by multivariate logistic regression analysis.

Variable	Unadjusted OR (95% CI)	*p*	Adjusted OR (95% CI)	*p*
BPD	2.29 (1.89–2.77)	<0.001	1.88 (1.48–2.40)	<0.001
Survival without severe complications	0.58 (0.51–0.67)	<0.001	0.75 (0.63–0.91)	0.003
Severe brain injury	0.75 (0.59–0.96)	0.022	0.62 (0.46–0.84)	0.002
Severe ROP	1.19 (1.06–1.34)	0.003	0.95 (0.81–1.12)	0.515
NEC	0.94 (0.77–1.15)	0.555	0.77 (0.60–1.01)	0.055
Death	1.04 (0.93–1.17)	0.515	0.66 (0.53–0.83)	<0.001

BPD, bronchopulmonary dysplasia; ROP, retinopathy of prematurity; NEC, necrotizing enterocolitis.

### 3.5 Characteristics of clinical outcomes in different transfusion strategy groups

To eliminate the confounding effect of critical illness severity on clinical outcomes and ensure the robustness of comparisons across transfusion strategies, the 346 transfused infants were further divided into critically ill neonates (*n* = 34) and non-critically ill neonates (*n* = 312). Subgroup analysis by transfusion strategy was performed only among the non-critically ill infants, yielding 82 in the restrictive transfusion group, 138 in the liberal transfusion group, and 92 in the relatively liberal transfusion group. The number of transfusions differed significantly among the three groups (*p* < 0.001). Pairwise comparisons revealed that the relatively liberal transfusion group had a significantly higher number of transfusions than the other two groups.

Although the overall incidence of BPD showed a marginally significant difference among the three groups (*p* = 0.047), no statistical significance was observed in pairwise comparisons after Bonferroni adjustment (all adjusted *p* ≥ 0.0167). Similarly, the rates of survival without severe complications, severe brain injury, severe ROP, NEC, and mortality were all comparable between groups (all *p* > 0.05). See Table 7 for additional details.

**Table 7:** Comparison of clinical outcomes among different transfusion strategy groups.

Variable	Restrictive transfusion (*n* = 82)	Liberal transfusion (*n* = 138)	Relatively liberal transfusion (*n* = 92)	*χ²/Z*	*p*
Number of transfusions, median (IQR), *n*	1 (1–2)^a^	1 (1–2)^a^	3 (2–4)^b^	122.14	<0.001
BPD, *n* (%)	28 (43.1)^a^ [*n* = 65]	35 (28.7)^a^ [*n* = 122]	38 (43.2)^a^ [*n* = 88]	6.10	0.047
Survival without severe complications, *n* (%)	27 (32.9)	63 (45.7)	31 (33.7)	4.93	0.085
Severe brain injury, *n* (%)	6 (7.3)	9 (6.5)	7 (7.6)	0.11	0.946
Severe ROP, *n* (%)	14 (19.7) [*n* = 71]	27 (22.0) [*n* = 123]	24 (27.0) [*n* = 89]	1.30	0.522
NEC, *n* (%)	11 (13.4)	13 (9.4)	9 (9.8)	0.96	0.620
Death, *n* (%)	19 (23.2)	17 (12.3)	12 (13.0)	5.20	0.074

a,b Different superscripts indicate statistically significant differences between groups after Bonferroni correction; the same superscript denotes no significant difference. The number of cases varies due to outcome-specific exclusion during data analysis. Denominators are as specified in column heads unless otherwise indicated.

BPD, bronchopulmonary dysplasia; ROP, retinopathy of prematurity; NEC, necrotizing enterocolitis.

## 4 Discussion

The primary purpose of transfusion is to improve oxygen-carrying capacity, particularly in infants with increased oxygen demand due to critical illness. However, studies have shown an association between transfusion and adverse outcomes (3, 6–10). The underlying pathological mechanisms involve multiple factors, including storage lesions, immune-inflammatory responses, oxidative damage, and the transfusion itself. Firstly, red blood cells undergo storage lesions during *in vivo* storage (20), manifesting as membrane damage, reduced deformability, microparticle formation, and accumulation of inflammatory factors. These changes can lead to immune dysregulation, vascular endothelial injury, tissue hypoxia, and other adverse effects. The products of storage lesions, together with residual leukocytes, contribute to transfusion-related immunomodulation (TRIM), triggering either proinflammatory or immunosuppressive effects (21). Secondly, frequent transfusions can lead to iron overload (22), which in turn induces oxidative damage and inflammatory responses (23). Furthermore, transfusion-related acute lung injury and transfusion-associated circulatory overload are common cardiopulmonary complications (24), which can cause pulmonary injury and circulatory overload, respectively. Bedford et al. (25) found that preterm infants receiving multiple transfusions of non-leukoreduced whole blood developed antihuman leukocyte antigen antibodies, whereas those receiving leukoreduced blood products did not. Fergusson et al. (26) reported that universal prestorage leukoreduction significantly decreased the risks of BPD, ROP, and NEC. Crawford et al. observed that early and repeated transfusion induced alterations in proinflammatory cytokines and markers of endothelial activation in very preterm infants and suggested that the potential for TRIM was present in the initial days after birth (27), and subsequent research indicated that transfusion of washed red blood cells might attenuate this response (28). These studies provide important evidence supporting the reduction of transfusion-related risks through the modification of blood products.

Given the long study period from 2012 to 2024, significant changes had occurred in neonatal comprehensive management strategies, including transfusion guidelines, respiratory support protocols, and overall care practices. To account for potential confounding introduced by temporal changes in clinical practice over the decade, we categorized the study period into two treatment eras (2012–2019 vs. 2020–2024) and included treatment era as a covariate in all multivariate regression models. After adjustment for confounders including treatment era, multivariate analysis demonstrated that an increased number of transfusions was an independent risk factor for BPD. Meanwhile, a higher transfusion count was independently associated with a lower risk of severe brain injury and mortality. Despite these inverse associations, the rate of survival without severe complications was significantly lower with an increasing number of transfusions.

Of note, the association between transfusion and severe brain injury was not significant in the overall cohort analysis, which may be attributable to reverse causality, as transfusions administered after the onset of brain injury could mask the true relationship. In contrast, the logistic regression models included only transfusions occurring before the diagnosis of severe brain injury, which substantially reduced reverse causality and time-dependent bias. After full adjustment, an increased number of transfusions was independently associated with a lower risk of severe brain injury, and this result differs from a previous study (9). A secondary study of the TOP trial by Chock et al. (29) revealed a significant transfusion-associated increase in cerebral tissue saturation (Csat) that was of comparable magnitude in both restrictive and liberal transfusion groups. Wang et al. (30) reported that the number of transfusions within 7 days of life was positively correlated with cognitive performance at 18–24 months of corrected age. Collectively, these findings suggest that transfusions may confer neuroprotective effects by ameliorating cerebral hypoxia.

Studies have confirmed that anemia (rather than transfusion itself) acts as an independent risk factor for ROP (19) and NEC (31). While observational studies reported increased risks of death and NEC associated with transfusions, randomized controlled trials found no such association (32, 33). This heterogeneity may originate from confounding factors like illness severity. The present study demonstrated that transfusion number was not independently associated with severe ROP or NEC. For mortality, no significant association was observed in the overall cohort or univariate analysis. However, after adjustment for confounders, an increased number of transfusions was independently associated with a reduced risk of mortality. This protective effect may be attributed to the ability of transfusion to correct life-threatening anemia, improve systemic oxygen delivery, and stabilize hemodynamic status.

Despite the reduced risks of mortality and severe brain injury, the rate of survival without severe complications remained decreased. This may be explained by the strong independent association between transfusion and BPD, which is a core component of the composite outcome. The increased risk of BPD associated with more transfusions, as confirmed by the present study and previous reports (6), may partially offset the benefits derived from the reduced risks of mortality and severe brain injury. Furthermore, previous studies (27) have confirmed that repeated transfusions may induce persistent inflammation and immune dysregulation, which may further increase the risk of other complications and ultimately contribute to the decreased rate of survival without severe complications. In this study, transfused infants had lower GA, lower BW, a higher proportion of critically ill infants (SNAPPE-II score ≥40), higher rates of pulmonary hemorrhage, sepsis, and hsPDA, longer hospital stay, and longer duration of respiratory support, all indicating greater underlying illness severity. Consequently, adverse outcomes more likely originate from underlying pathology than transfusions, rendering the observed transfusion-outcome relationship associative rather than causal.

The association between restrictive vs. liberal transfusion strategies and clinical outcomes also remains controversial across studies. The Iowa study (34) demonstrated a higher incidence of grade 4 IVH or PVL and apnea in the restrictive transfusion group, suggesting a potential increased risk of neurological injury with restrictive transfusion. However, long-term follow-up revealed significantly smaller intracranial volume in the liberal transfusion group compared to the restrictive transfusion group (35), indicating possible long-term adverse effects of the liberal strategy. Despite a lower incidence of cognitive delay in the liberal transfusion group, which provided weak evidence for a potential neurodevelopmental benefit, no differences were observed in short-term morbidity (death, BPD, severe ROP, brain injury) or the long-term composite outcome of death or NDI in the PINT trial (36) and its follow-up study (37). The more representative TOP (4) and ETTNO (11) trials also showed no significant differences in short- or long-term adverse outcomes between restrictive and liberal transfusion groups. Although some infants in the restrictive transfusion group received transfusions above the threshold due to clinical needs (e.g., major bleeding, severe hypoxemia), this did not affect overall outcomes, highlighting the necessity for individualized decision-making. Crucially, the analysis by Chock et al. (29) further identified that the number of transfusions with mean pretransfusion Csat <50% was associated with NDI or death. This indicates that transfusion threshold itself did not directly influence outcomes, but rather that a lower pretransfusion Csat may be associated with adverse outcomes, supporting further investigation of targeted tissue saturation monitoring in preterm infants with anemia. A meta-analysis (38) indicated that liberal transfusion threshold significantly increased the level of Hb after transfusion and reduced the duration of supplemental oxygen and ventilator or continuous positive airway pressure, potentially related to reduced supplemental oxygen needs with higher Hb levels; however, no differences in short- or long-term adverse outcomes were found. In our study, the restrictive transfusion group had significantly fewer transfusions, with no significant between-group differences detected in BPD, survival without severe complications, severe ROP, NEC, severe brain injury, or death. Synthesizing the current evidence, the liberal strategy demonstrates no clear clinical advantage, whereas the restrictive strategy reduces the number of transfusions and donor exposure risks. This benefit has made the restrictive strategy the prioritized approach in current international guidelines.

This study referenced the transfusion thresholds recommended by the Neonatal Transfusion Network guideline (12), which was developed based on the TOP and ETTNO trials. While these two trials differed in their definitions for higher level of illness severity (any respiratory support or critical state of health), the steering committee unified these criteria. They defined higher level of illness severity as requiring mechanical ventilation, continuous positive airway pressure, noninvasive intermittent positive pressure ventilation, or nasal cannula flow rate ≥1 liter per minute. This study involved 798 transfusion episodes among 545 infants, of which 363 episodes (45.5%) were administered below the recommended threshold. All transfusions during the first two weeks postpartum required varying degrees of respiratory support, categorizing these infants under a “higher level of illness severity” per guideline definitions. Transfusions without or minimal respiratory support occurred exclusively at ≥3 weeks postpartum. However, only 1 case (0.6%) of threshold-below transfusion was documented in this subgroup, indicating substantial room for improvement in transfusion management.

This study also has several limitations. First, as a single-center retrospective study, it is susceptible to selection bias. Second, the sample sizes were imbalanced among the three transfusion strategy groups (restrictive, liberal, and relatively liberal), which may reduce statistical power and affect the precision of comparisons. Additionally, the lack of long-term follow-up data limits the assessment of long-term outcomes. Fourth, transfusion strategies were classified retrospectively based on *post-hoc* threshold definitions rather than randomization, which may introduce additional bias. Future studies should employ multicenter prospective designs incorporating both short- and long-term outcomes. By integrating monitoring techniques such as near-infrared spectroscopy (NIRS) for measuring Csat, we aim to balance immediate benefits against long-term risks, thereby exploring personalized transfusion strategies to enhance the quality of life in preterm infants.

## 5 Conclusions

In summary, the number of transfusions is an independent risk factor for BPD and is independently associated with a lower likelihood of survival without severe complications in ELGANs. In non-critically ill ELGANs, a restrictive transfusion strategy reduces transfusion exposure without increasing the risk of adverse outcomes. Therefore, the use of a restrictive transfusion strategy is recommended in clinical practice, with individualized adjustments based on the specific clinical condition of each infant.

## Data Availability

The raw data supporting the conclusions of this article will be made available by the authors, without undue reservation.
